# Primary hydatid cyst in the axillary region: A case report

**DOI:** 10.1016/j.ijscr.2025.110919

**Published:** 2025-01-22

**Authors:** Lama Kanaa, Fatima Breim, Abdalwahab Alkhalf, Mousa Sifat, Ahmad Ghazal

**Affiliations:** a Faculty of Medicine, University of Aleppo, Aleppo, Syria; b Department of General Surgery, Faculty of Medicine, University of Aleppo, Aleppo, Syria

**Keywords:** Hydatid cyst, Echinococceal cyst, Echinococcosis, Axilla, Case report

## Abstract

**Introduction:**

Hydatid disease, caused by the larval stage of Echinococcus granulosus, is a significant zoonotic infection predominantly affecting the liver and lungs. While hydatid cysts are commonly found in internal organs, cases in the axillary region are rare.

**Presentation of case:**

We report a unique case of a 52-year-old female patient presenting with a painless left axillary swelling for two years. Physical examination revealed a firm, mobile mass measuring 10 × 5 cm, with no associated lymphadenopathy. Laboratory tests indicated normal results, while ultrasound imaging confirmed a thick-walled cystic lesion. The patient underwent total cystectomy under general anesthesia, and histopathological analysis confirmed the diagnosis of a hydatid cyst.

**Discussion:**

Hydatid cysts typically originate in the liver or lungs, with axillary primary cysts being rarely documented, with less than 20 prior cases in English literature. The mechanism for larvae migration to the axillary region remains unclear. The patient exhibited a mobile, asymptomatic mass, and imaging studies were crucial for diagnosis, emphasizing that differential diagnoses should include various axillary masses such as lymphadenitis or neoplasms.

**Conclusion:**

This case highlights the need for awareness of axillary hydatid cysts in endemic regions, which may be misdiagnosed due to their rarity. Prompt diagnosis and individualized treatment, including total cystectomy and adjunctive medical therapy with albendazole, are critical to prevent complications and recurrence of hydatid disease.

## Highlights

• Echinococcus granulosus causes hydatid disease, a zoonotic infection.
• Hydatid cysts typically form in the liver and lungs, rarely in other organs.
• Axillary hydatid cysts are extremely rare, even in endemic regions.
• This report highlights the importance of considering hydatid cysts in the differential diagnosis of palpable axillary masses.

## 1 Introduction

Hydatid disease is a parasitic infection caused by the larval stage of Echinococcus granulosus, a small zoonotic tapeworm primarily associated with dogs, while sheep, cattle, and deer serve as intermediate hosts. Humans are considered incidental hosts in this lifecycle, becoming infected by ingesting eggs present in soil or water that has been contaminated by infected dog feces [1]. This disease is endemic in many countries, particularly in the Mediterranean region, Australia, South America, the Middle East, South Africa, and Eastern Europe [2].

Typically, the majority of patients (40–80 %) present with a solitary cystic lesion localized to a single organ, with the liver being involved in approximately 70 % of these cases. The lungs are the second most commonly affected organ, occurring in about 20 % of instances. While hydatid cysts can develop in virtually any organ or structure—including the brain, heart, orbit, urinary bladder, chest wall, subcutaneous tissue, tibia, parotid gland, breast, cervicofacial region, and thyroid—axillary hydatid cysts are extremely rare [1,3].

Although hydatid cysts are often asymptomatic, the symptoms can vary widely based on the size and location of the cyst. Complications, such as cyst rupture, can lead to severe outcomes, including secondary infections or anaphylactic reactions [1].

In this case report, we present a unique instance of a hydatid cyst located in the axillary region of a 52-year-old female patient. This case is particularly noteworthy due to the rarity of axillary hydatid cysts and the potential for clinical misdiagnosis with other types of axillary masses. Given the infrequent occurrence of hydatid cysts in this anatomical location, accurately distinguishing them from other conditions presents a significant challenge that can impact treatment decisions. Our aim is to highlight this unusual presentation and discuss its implications for clinical practice.

The work has been reported in line with the SCARE criteria [4].

## 2 Presentation of case

**Fig. 1:**
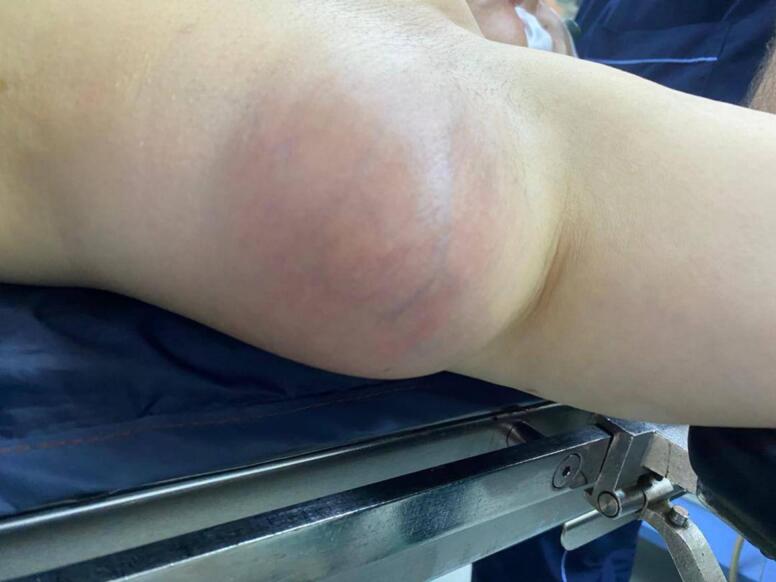
Preoperative photograph showing a large mass of the left axillary region.

A 52-year-old woman presented to our hospital with a painless left axillary swelling persisting for two years. Fig. 1. The patient reported no history of breast masses, fever, or other symptoms and had no previous diagnosis of hydatid cysts.

On examination, a firm to hard, mobile mass measuring (10 × 5) cm was palpated in the left axilla. There was no evidence of lymphadenopathy or noticeable changes in the overlying skin.

Laboratory investigations revealed normal blood counts and biochemical results, including liver function tests and inflammatory markers.

Ultrasound imaging demonstrated a (10 × 5) cm thick-walled cystic lesion. The nature of the cyst was further elucidated, noting that it appeared anechoic without internal echoes. A mammographic examination of both breasts showed no abnormalities.

Preoperative imaging studies were conducted to evaluate potential involvement of other organs, including abdominal ultrasound and chest X-ray, which showed no signs of liver or lung involvement. Based on characteristic ultrasound findings, the lesion was suspected to be a hydatid cyst.

**Fig. 2:**
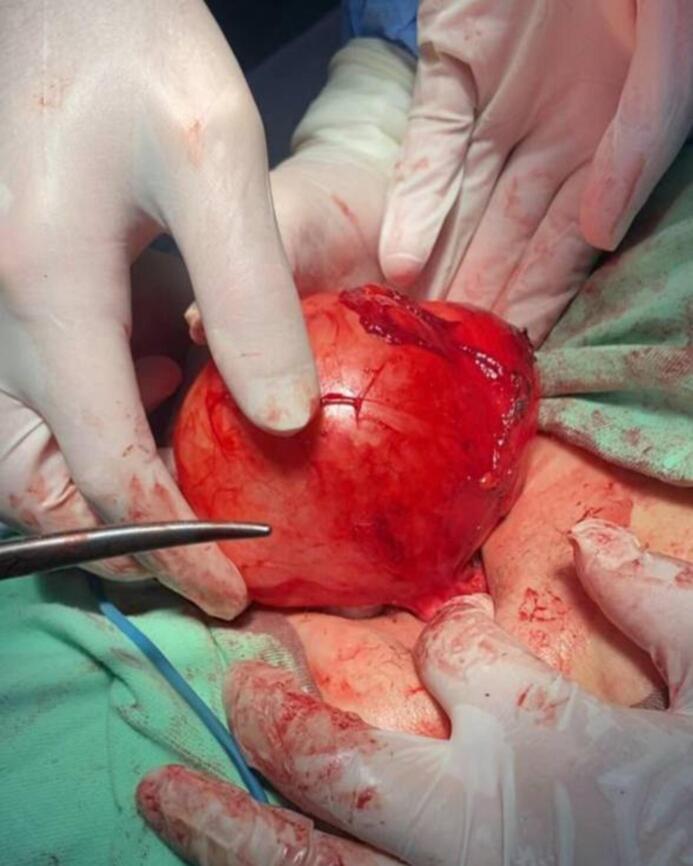
Operation of cystic mass lesion at axillary area.

Under general anesthesia, total cystectomy was performed. The cyst was carefully excised without rupture to minimize the risk of intraoperative dissemination. The exocyst was excised to ensure complete removal of all cyst components, which is crucial for preventing recurrence. Fig. 2.

**Fig. 3:**
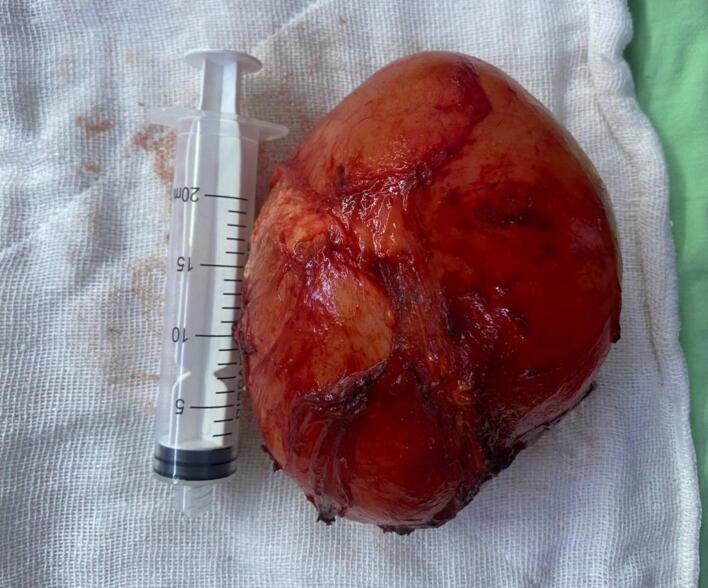
Grossly, non-ruptured cyst, with a hard fibrotic wall, measuring (12 × 10 × 6) cm.

**Fig. 4:**
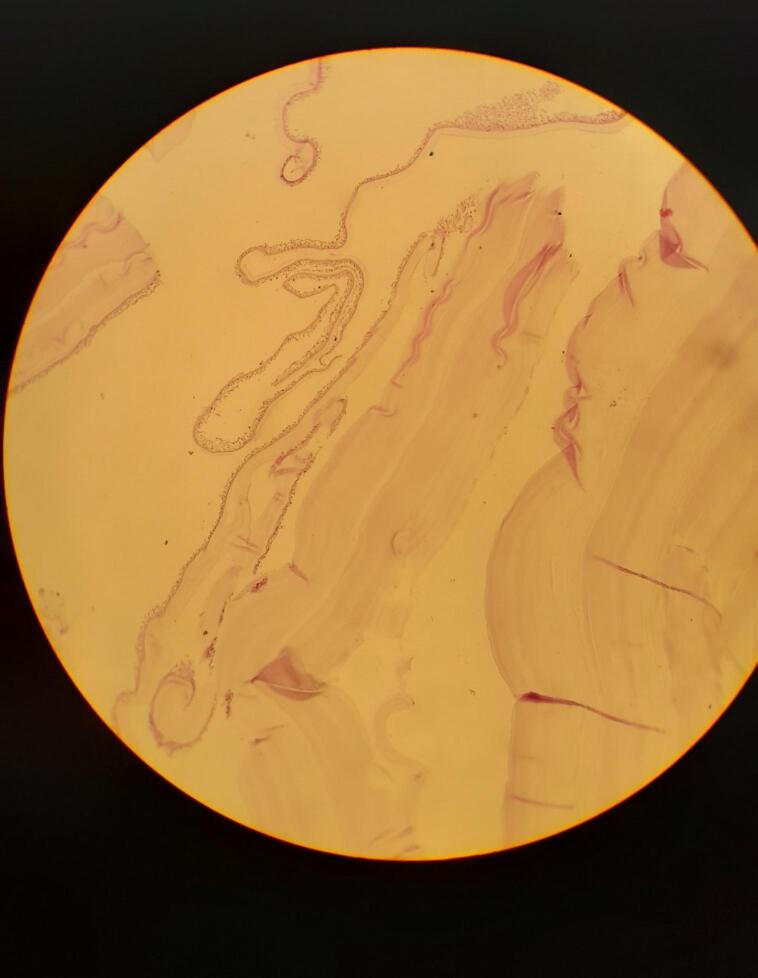
Microscopic examination: Section show acellular double-layered laminated capsule with inner cell monolayer (germinal membrane) and the presence of congested fibrovascular wall surrounded by a reactive fibrous capsule.

Macroscopic examination revealed a unilocular cyst with a hard fibrotic wall, measuring (12 × 10 × 6) cm. Fig. 3. Microscopic examination confirmed the diagnosis of a hydatid cyst, revealing the typical laminated structure associated with the infection, characterized by an acellular double-layered laminated capsule with inner cell monolayer (germinal membrane) and the presence of congested fibrovascular wall surrounded by a reactive fibrous capsule. (Fig. 4).

The postoperative course was uneventful. The patient was started on a regimen of albendazole at a dosage of 400 mg twice daily for four weeks to limit the risk of intraoperative dissemination of daughter cysts.

## 3 Discussion and conclusion

Hydatid cyst disease is a widespread public health problem in developing country. The parasite, Echinococcus granulosis, belongs to the family Taeniidae and human can serve as accidental intermediate hosts in its life cycle [5].

The majority of hydatid cysts are typically found in the liver (50–60 %) and lungs (20–30 %). In 13.9 % of the cases, hydatid cyst is found in uncommon location [6].

Primary hydatid cysts in the axillary region are rare, even in areas where it is endemic, with less than 20 prior cases in English literature [7]. The average age of the patients involved is 42.5 years, and the likelihood of infection is the same for both males and females [2].

The precise mechanism by which larvae reach the axillary region remains unclear. However, several hypotheses can be proposed regarding the formation of cysts in this area. The larvae may bypass the gastrointestinal tract and enter either the hepatic or pulmonary circulation, subsequently entering the bloodstream or lymphatic system to reach the axillary region. Additionally, some studies have suggested that infection of a muscle within the axillary cavity may occur following direct exposure to a dog bite, facilitating the proliferation of larvae in this specific area [7,8].

In summary, these hypotheses explore the possible pathways for echinococcosis larvae to develop into hydatid cysts in the axillary region, and there is no evidence regarding how our patient was infected.

Our case demonstrates primary involvement of the axillary region without any signs of disease in the internal organs to rule out the possibility of larval migration.

The main symptoms include unusual clinical features such as swelling, pain, a mobile palpable mass, pruritus, and a sense of malaise, and may present with no symptoms at all [2]. Our patient presented with an asymptomatic mobile palpable mass in her left axilla.

Imaging techniques such as ultrasound, computed tomography (CT), and magnetic resonance imaging (MRI) can effectively assess the relationship of cysts to adjacent tissues, as well as the inner wall and any daughter cysts present within. Routine laboratory tests (e.g. complement fixation and enzyme-linked immunosorbent assay) typically do not reveal any specific finding related to hydatid cysts and they have high false positivity rates [6]. Total cystectomy was planned to diagnose and manage the mass lesion in the axilla, rather than proceeding with additional imaging studies and this was confirmed through macroscopic and microscopic analysis of the specimen in our case.

Nonetheless, it should be considered in the different diagnoses of a palpable painless mass, such as lymphadenitis, abscess, soft tissue sarcomas, lymphocele, or metastasis breast cancer [7]. Differential diagnoses involve a thorough assessment, including medical and travel history, physical exams, imaging (ultrasound, CT, MRI), serological tests, and histopathological analysis of biopsies and tissues with specific stains.

Management options and treatment should be individualized on a patient-to-patient basis, including surgery, PAIR (puncture, aspiration, injection, and respiration), and radical surgical excision [2]. The most effective method for treating is cystectomy without rupture to prevent the likelihood of recurrent infestation [2]. Total cystectomy without rupture of the cyst or medical treatment was done in our case.

Medical treatment involves the use of mebendazole and albendazole, particularly for cases of widespread or hard-to-reach hydatidosis, as well as for patients who prefer to avoid the complications of surgery [9]. Our patient receive albendazole at a dosage of 400 mg twice daily for four weeks to reduce the viability of any remaining cysts and minimize the risk of spreading viable daughter cysts during surgical intervention.

In conclusion, while primary axillary hydatid cysts are very rare, they should be included in the differential diagnosis for patients presenting with axillary swelling in endemic locations. Awareness among healthcare providers about this potential condition can facilitate prompt treatment and reduce complications associated with hydatid disease.

## Consent for publication

Written informed consent was obtained from the patient for publication of this case report and accompanying images. A copy of the written consent is available for review by the Editor-in-Chief of this journal on request.

## Ethical approval

This study is exempt from ethical approval in our institution (Aleppo University Hospital, Faculty of Medicine, University of Aleppo, Aleppo, Syria) because the content of the single case report does not require ethical approval.

## Funding

This research did not receive any specific grant from funding agencies in the public, commercial, or not-for-profit sectors.

## Author contribution

Ahmad Ghazal supervised and helped in writing the manuscript. Lama Kanaa, Fatima Breim, Abdalwahab Alkhalf and Mousa Sifat wrote the manuscript. Lama Kanaa, critically revised the manuscript. All authors read and approved the final manuscript.

## Guarantor

Lama Kanaa.

## Research registration number

Not applicable.

## Declaration of competing interest

None.

## Data Availability

All data generated or analyzed during this study are included in this published article and its supplementary information files.
